# Reported muscle symptoms during statin treatment amongst Italian dyslipidaemic patients in the real‐life setting: the PROSISA Study

**DOI:** 10.1111/joim.13219

**Published:** 2020-12-29

**Authors:** M. Casula, M. Gazzotti, F. Bonaiti, E. OImastroni, M. Arca, M. Averna, A. Zambon, A.L. Catapano, the PROSISA Study Group, Marcello Arca, Anna Montali, Maurizio Averna, Antonina Giammanco, Gianni Biolo, Pierandrea Vinci, Claudio Borghi, Sergio D’Addato, Antonio Carlo Bossi, Giancarla Meregalli, Adriana Branchi, Giovanna Squiccimarro, Franco Cavalot, Linda Ramadori, Francesco Cipollone, Marco Bucci, Maria Del Ben, Francesco Angelico, Anna Maria Fiorenza, Emanuela Colombo, Liliana Grigore, Veronica Zampoleri, Graziana Lupattelli, Vito Gandolfo, Giuseppe Mandraffino, Francesca Savarino, Giuliana Mombelli, Chiara Pavanello, Livia Pisciotta, Andrea Pasta, Francesco Purrello, Roberto Scicali, Paolo Rubba, Giuliana Fortunato, Carlo Sabbà, Patrizia Suppressa, Riccardo Sarzani, Chiara Di Pentima, Giovanni Battista Vigna, Angela Colangiulo, Josè Pablo Werba, Lorenzo Maria Vigo, Sabina Zambon, Lorenzo Previato, Maria Grazia Zenti, Chiara Maneschi

**Affiliations:** ^1^ Epidemiology and Preventive Pharmacology Service (SEFAP) Department of Pharmacological and Biomolecular Sciences University of Milan Milan Italy; ^2^ IRCCS MultiMedica Milan Italy; ^3^ Department of Translational and Precision Medicine Unit of Internal Medicine and Metabolic Diseases Sapienza University Rome Italy; ^4^ Department of Health Promotion Sciences Maternal and Infantile Care Internal Medicine and Medical Specialities University of Palermo Palermo Italy; ^5^ Department of Medicine‐DIMED University of Padua Padua Italy

**Keywords:** adverse effects, myopathy, statin‐associated muscle symptoms, statins

## Abstract

**Aim:**

Statin‐associated muscle symptoms (SAMS) are a major determinant of poor treatment adherence and/or discontinuation, but a definitive diagnosis of SAMS is challenging. The PROSISA study was an observational retrospective study aimed to assess the prevalence of reported SAMS in a cohort of dyslipidaemic patients.

**Methods:**

Demographic/anamnestic data, biochemical values and occurrence of SAMS were collected by 23 Italian Lipid Clinics. Adjusted logistic regression was performed to estimate odds ratio (OR) and 95% confidence intervals for association between probability of reporting SAMS and several factors.

**Results:**

Analyses were carried out on 16 717 statin‐treated patients (mean ± SD, age 60.5 ± 12.0 years; 52.1% men). During statin therapy, 9.6% (*N* = 1599) of patients reported SAMS. Women and physically active subjects were more likely to report SAMS (OR 1.23 [1.10–1.37] and OR 1.35 [1.14–1.60], respectively), whist age ≥ 65 (OR 0.79 [0.70–0.89]), presence of type 2 diabetes mellitus (OR 0.62 [0.51–0.74]), use of concomitant nonstatin lipid‐lowering drugs (OR 0.87 [0.76–0.99]), use of high‐intensity statins (OR 0.79 [0.69–0.90]) and use of potential interacting drugs (OR 0.63 [0.48–0.84]) were associated with lower probability of reporting SAMS. Amongst patients reporting SAMS, 82.2% underwent dechallenge (treatment interruption) and/or rechallenge (change or restart of statin therapy), with reappearance of muscular symptoms in 38.4% (3.01% of the whole cohort).

**Conclusions:**

The reported prevalence of SAMS was 9.6% of the whole PROSISA cohort, but only a third of patients still reported SAMS after dechallenge/rechallenge. These results emphasize the need for a better management of SAMS to implement a more accurate diagnosis and treatment re‐evaluation.

## Graphical abstract

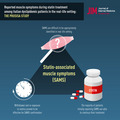

## Introduction

Statins are the cornerstone of pharmacological therapy for low‐density lipoprotein cholesterol (LDL‐C) lowering and play a pivotal role in the prevention and treatment of coronary heart disease and other cardiovascular (CV) diseases [1]. Although statins are generally safe and well tolerated, not all patients are able to use them at the recommended dose [2]. Indeed, all statins are associated with some adverse effects (AE) including muscle‐related AE, hepatic or gastrointestinal AE, especially at higher doses, which could lead patients to discontinue or interrupt the statin treatment [3]. The problem of ‘statin intolerance’, defined as the inability to tolerate a dose of statin required to appropriately reduce a patient’s CV risk, represents a relevant health issue [4], particularly in secondary prevention.

The most frequent cause of intolerance is represented by statin‐associated muscle symptoms (SAMS) [5]. Almost 30% of subjects on statin therapy complain of muscle symptoms [5], and SAMS are reported as the primary reason for statin nonadherence and discontinuation by 60% of patients who interrupt the therapy [6, 7]. SAMS include myalgia, myopathy, myositis and muscle injury in some instances leading to rhabdomyolysis. Defining SAMS is further compounded by no current consensus on the terminology to be used, because the terms myalgia, myositis and myopathy are often interchanged and misused [5]. Moreover, increased plasma levels of creatine kinase (CK), a biomarker of muscle damage, are not consistently associated with SAMS [8], imaging methods for the detection of SAMS are missing, and the electromyogram provides no specific findings with SAMS even with increased CK plasma levels [8]. Moreover, the absence of a univocal definition of statin intolerance [9], together with the lack of a ‘gold standard’ diagnostic test and of a validated, universally accepted, questionnaire for the evaluation of SAMS, makes it difficult to estimate the real incidence rate of this event.

In the absence of a unique definition, Consensus documents by European and Canadian Groups [5, 10] proposed to define SAMS according to the nature of symptoms and their temporal relationship with statin initiation. Moreover, withdrawal (dechallenge) and re‐exposure (rechallenge) to statin treatment have been suggested by current guidelines as useful tool to screen whether reported muscle symptoms are truly associated with statin medication. Usually, after this approach, the diagnosis of SAMS is confirmed only in 5%–6% of patients [5, 11]. This percentage further decreases to 2%–3% if a gradual diagnostic approach is applied, which includes the following: (i) meticulous physical examination of the patient, (ii) evaluation of previous history and (iii) evaluation of drug interactions and exclusion of possible risk factors for SAMS, including the nocebo effect [12], which is associated with the person's prior expectations of adverse effects from treatment as well as with conditioning in which the person learns from prior experiences to associate a medication with certain somatic symptoms or from observations of symptoms in other patients. Indeed, available data show that the reported incidence of SAMS is consistently lower in randomized controlled trials (RCTs) than in observational studies [13, 14]. This evidence, combined with the absence of a univocal definition and the heterogeneity of the clinical presentation, makes it challenging to estimate the incidence of this phenomenon, especially in a real‐world context. Therefore, the main aim of the PROSISA (PROject Statin Intolerance SISA) study was to assess the prevalence of statin intolerance, due to muscular symptoms, in a cohort of dyslipidaemic patients on statin therapy in a real‐life setting.

## Methods

The PROSISA study was an observational, multicentre and retrospective study.

The study was approved by the Ethics Committee of the Coordinating Centre (Centro per lo studio dell’Aterosclerosi IRCCS Multimedica, Sesto San Giovanni, Milan, Italy, 24.05.2016) and then by the local Ethics Committees of each participating centre. It was conducted in accordance with the protocol, the standards of Good Clinical Practice (ICH GCP), the ethical principles of Helsinki Declaration, the data protection laws and other applicable regulations.

### Study population

The study included subjects ≥ 18 years, of both sexes managed in one of the 23 Italian Lipid Clinics participating in the Italian Atherosclerosis Society (SISA) network and reference centres for the diagnosis and treatment of dyslipidaemias (Figure S1). To be enrolled, hypercholesterolaemic patients should have been treated with any statins at any dosage in a period between 1 January 2006 and 31 December 2015 (index visit) and followed up by one of the SISA centres involved in the study.

### Data collection

The data collection was performed using an electronic case report form (eCRF) in a strictly anonymous way to guarantee the privacy of every patient. Because of the retrospective and observational nature of the study, patients did not undergo any procedures other than normal clinical practices, and only clinical variables collected in daily practice by physicians were recorded in the eCRF.

For each enrolled subject, demographic data, height and weight, blood pressure, lifestyle habits and pathological anamnesis at index visit were collected. As far as statin treatment is the concern, type and dose of statin, other lipid‐lowering therapies, lipid profile pre‐ and on‐treatment, and concomitant use of drugs known for their potential interaction with statins were collected. Moreover, muscle symptoms reported by subjects, as annotated in clinical records by physicians, and time to symptom onset since starting statin treatment were collected in eCRF as well.

For patients reporting muscular symptoms, the study protocol required to report data about two additional phases: dechallenge (statin interruption) and rechallenge (statin reintroduction, or dose reduction if the dechallenge was not applied), if performed and available. The dechallenge phase consisted of a period of complete statin withdrawal, and data about the lipid profile and persistence of muscular symptoms were collected, if available. For patients who had their statin treatment re‐administered, data about the rechallenge phase were also collected. According to routine clinical approach, some SAMS‐reporting subjects did not interrupt statin treatment, as for to physician’s judgement, and changed statin treatment without any wash‐out period; for these patients, rechallenge data were collected even in the absence of a dechallenge phase. In particular, for these patients, data about the new treatment, the lipid profile and the recurrence of muscular symptoms were collected, as shown in Figure S2.

In addition, a survey was carried out in order to evaluate the different approaches used by centres in the diagnosis and management of SAMS. The questionnaire consisted of multiple‐choice questions regarding the routine clinical practice approach in the evaluation of the SAMS diagnostic criteria. The presence of SAMS was considered confirmed in patients with dechallenge and rechallenge phases, that is, if the muscle symptoms reported at the index visit were not reported during treatment interruption and then were reported again with statin reintroduction. For patients who did not perform the dechallenge phase, SAMS were confirmed if the muscle symptoms at the index visit did not disappear after dose reduction or change in statin.

### Statistical analysis

Continuous variables are expressed as mean ± standard deviation (SD), whilst categorical variables are expressed as cases (*N*) and percentage rate (%). Chi‐square or ANOVA for categorical variables with more than two modalities and t‐tests were used to evaluate the differences in categorical and continuous variables between groups, respectively.

A multivariate logistic regression model was performed to estimate odds ratios (OR) and 95% confidence intervals [95% CI] for the association between the probability of reporting SAMS and several covariates included sex, age (≥65 years or younger), body mass index (BMI, classified as <25, 25–29.9, ≥30 kg m^−2^), physical activity (yes/no), familial dyslipidaemias (yes/no), hypertension (yes/no), type 2 diabetes mellitus (yes/no), previous CV event (yes/no), concomitant lipid‐lowering treatments (yes/no) and statin intensity (low/high) [15].

Because data on some covariates were missing in some patients, the corresponding analyses were performed by allocating missing values in a separate category. The model was adjusted for centres, in order to minimize heterogeneity.

Statistical significance was set at the 0.05 level for each analysis performed. All analyses were performed using the Statistical Analysis System Software version 9.4 (SAS. Institute, Inc. Cary, NC).

This research was done without patient involvement. Patients were not invited to comment on the study design and were not consulted to develop patient‐relevant outcomes or interpret the results. Patients were not invited to contribute to the writing or editing of this document for readability or accuracy.

## Results

### Baseline

Our study cohort included 16 717 valid subjects, with mean age of 60.5 years (±12.0 years, 58.9 [±12.6] in men and 62.9 [±11.2] in women, *P* < 0.001), and about 52.1% of them were men (Table 1). At baseline, 18.6% of the cohort was current smoker, and 24.4% reported regular physical activity. More than half of the cohort was hypertensive, and about one out of four had type 2 diabetes mellitus. Overall, 38.0% of subjects had a previous cardiovascular event. Pretreatment mean LDL‐C levels were 191.7 (±54.6) mg dL^−1^ [5.0 (±1.4) mmol L^−1^], whilst mean levels reported during statin treatment were 111.1 (±38.3) mg dL^−1^ [2.9 (±1.0) mmol L^−1^]. Simvastatin and atorvastatin were the most commonly prescribed statins (31.9% and 31.8%, respectively) (see Table 1). A concomitant nonstatin lipid‐lowering drug was prescribed to 25.3% of patients. In particular, ezetimibe and omega‐3 fatty acids were the most prescribed lipid‐lowering therapies (55.3% and 43.6%, respectively), whilst fibrates were prescribed in 10.7% of the cohort. The concomitant use of potential interacting drugs involved 6.5% of patients; amlodipine, diltiazem and amiodarone being the most frequently reported drugs.

**Table 1:** Characteristics of the PROSISA cohort

Males, *N* (%)	8699 (52.1%)
Age [years], mean (SD)	60.5 (12.0)
BMI [kg m^−2^], mean (SD)	27.3 (4.5)
Systolic blood pressure [mmHg], mean (SD)	131.3 (16.4)
Diastolic blood pressure [mmHg], mean (SD)	78.8 (9.7)
Physical activity, *N* (%)	2602 (24.4%)
Smoking status, *N* (%)	
Never	7630 (52.2%)
Former	4277 (29.2%)
Current	2720 (18.6%)
Hypertension, *N* (%)	8345 (57.3%)
Type 2 diabetes mellitus, *N* (%)	3439 (24.4%)
Any previous cardiovascular event, *N* (%)	5234 (38.0%)
Ischaemic heart disease, *N* (%)	2581 (19.5%)
Peripheral arterial disease, *N* (%)	3297 (24.5%)
Chronic kidney disease, *N* (%)	1165 (9.0%)
Significant liver disease, *N* (%)	765 (6.1%)
Total cholesterol [mg dL^−1^], mean (SD) [mmol L^−1^, mean (SD)]	
Pretreatment	281.6 (60.2) [7.3 (1.6)]
On‐treatment	192.1 (44.4) [5.0 (1.1)]
LDL cholesterol [mg dL^−1^], mean (SD) [mmol L^−1^, mean (SD)]	
Pretreatment	191.7 (54.6) [5.0 (1.4)]
On‐treatment	111.1 (38.3) [2.9 (1.0)]
HDL cholesterol [mg dL^−1^], mean (SD) [mmol L^−1^, mean (SD)]	
Pretreatment	54.3 (16.4) [1.4 (0.4)]
On‐treatment	53.8 (16.0) [1.4 (0.4)]
Triglycerides [mg dL^−1^], median (IQR) [mmol L^−1^, median (IQR)]	
Pretreatment	147.0 (102.0–225.0) [1.7 (1.2–2.5)]
On‐treatment	118.0 (86.0–166.0) [1.3 (1.0–1.9)]
Statin treatment at baseline, *N* (%; mean dose [mg day^−1^])	
Simvastatin	5285 (31.9%; 21.2)
Atorvastatin	5278 (31.8%; 20.6)
Rosuvastatin	4522 (27.2%; 12.1)
Pravastatin	856 (5.2%; 27.3)
Fluvastatin	419 (2.5%; 75.5)
Lovastatin	234 (1.4%; 21.2)
High‐intensity statin therapy, *N* (%)	4337 (26.2%)
Other lipid‐lowering treatments, *N* (%)	4218 (25.2%)
Potential statin interacting drugs, *N* (%)	1091 (6.5%)

BMI, body mass index; IQR, interquartile range.

### Reported SAMS

**Fig. 1:**
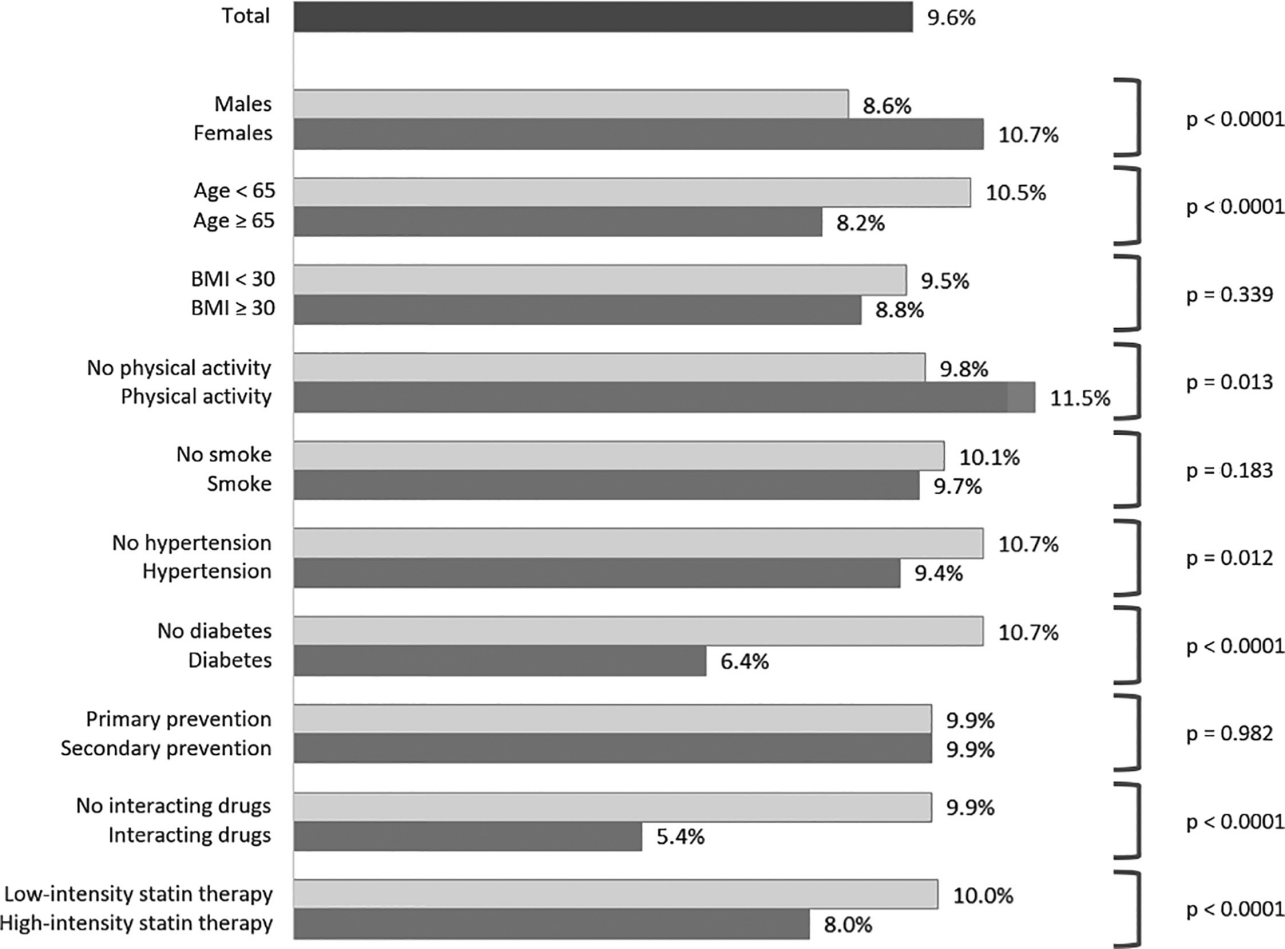
Percentage of patients reporting SAMS in the total cohort and in subgroups.

SAMS were reported by 9.6% (*N* = 1599) of the cohort (Fig. 1). This percentage was higher amongst women (10.7% vs. 8.7% in men, *P* < 0.0001) and in younger subjects (10.5% vs. 8.2% in subjects ≥ 65 years, *P* < 0.0001). Higher percentages of SAMS were also reported amongst subjects reporting physical activity (11.5% vs. 9.8% in inactive subjects, *P* = 0.0127). No differences in percentage of reported SAMS were detected between primary and secondary prevention, neither considering any previous cardiovascular event (9.9% in both groups, *P* = 0.982; Fig. 1) nor specifically in subjects with or without peripheral arterial disease (10.0% vs. 9.8, respectively, *P* = 0.711).

Amongst 1812 subjects with the information on plasma CK levels available at baseline, the percentages of reported SAMS were 10.8%, 12.7% and 19.4% in the three CK tertiles, respectively (*P* < 0.001). No significant difference was found on pretreatment LDL‐C levels between subjects reporting or not‐reporting SAMS: 194.5 (±55.5) mg dL^−1^ [5.0 (±1.4) mmol L^−1^] vs. 191.3 (±54.5) mg dL^−1^ [4.9 (±1.4) mmol L^−1^], respectively; *P* = 0.110).

Overall, 47.4% of reported SAMS occurred within 6 months since the initiation of statin therapy, and 25.3% of these within 3 months (Figure S3).

Amongst 1252 subjects who reported SAMS and with on‐treatment CK data available, 47.3% showed CK levels higher than ULN (upper limit of the normal range), and 5.1% of patients exhibited an increase in CK value more than 4xULN.

**Fig. 2:**
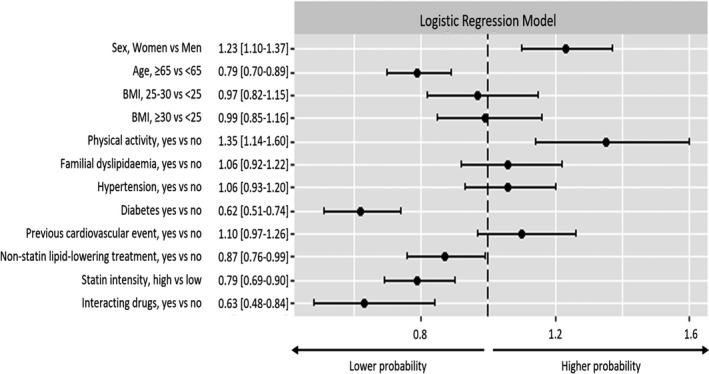
Adjusted odds ratio estimates for the probability of reporting SAMS (model adjusted for centre).

According to the results of the regression model (Fig. 2), women and subjects who were engaged in regular physical activity were more likely to report SAMS (OR 1.23; CI 95% 1.10–1.37; and OR 1.35; CI 95% 1.14–1.60, respectively), whilst older patients (OR 0.79; CI 95% 0.70–0.89), the presence of type 2 diabetes mellitus (OR 0.62; CI 95% 0.51–0.74), use of concomitant nonstatin lipid‐lowering drug (OR 0.87; CI 95% 0.76–0.99), prescription of high‐intensity statins (OR 0.79; CI 95% 0.69–0.90) and use of potential interacting drugs (OR 0.63; CI 95% 0.48–0.84) were associated with a low probability of reporting SAMS.

### SAMS confirmed by dechallenge/rechallenge

**Fig. 3:**
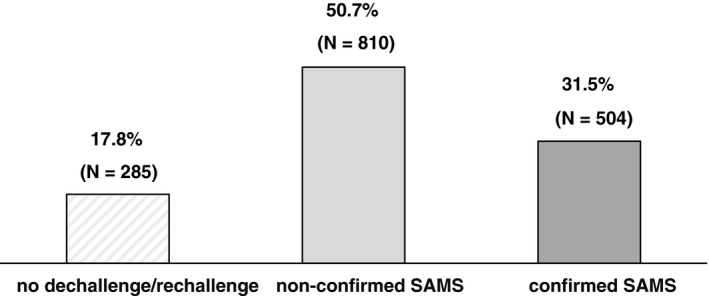
Evaluation of 1599 patients after reporting SAMS at the index visit, leading to a confirmation or not of SAMS through dechallenge/rechallenge phases.

As retrospectively obtained from clinical charts, 1599 patients reported SAMS. Of these, 1314 (82.2%) underwent dechallenge/rechallenge, whilst 285 (17.8%) did not follow this approach. Amongst patients undergoing dechallenge/rechallenge, 504 (38.4%) still reported SAMS. This group represents 31.5% of all patients reporting SAMS and 3.01% of the whole PROSISA cohort (Fig. 3).

The distribution of patients with confirmed SAMS through dechallenge/rechallenge as compared with those with nonconfirmed SAMS was similar by gender, age, presence of hypertension, type 2 diabetes mellitus and history of previous CV events. On the other hand, physical activity and use of drugs potentially interacting with statins were features more commonly found in patients with nonconfirmed SAMS (Table S1).

### SAMS management by lipid specialists

Results of the questionnaire underlined that only 26% of the lipidologists routinely warned patients of the possibility of muscle symptoms in normal clinical practice, whilst all are aware of the potential nocebo nature of this effect. In case of patients reporting SAMS, 83% of lipidologists reported that they measure CK routinely; 78% recognized that SAMS can occur even in the absence of increased CK.

**Fig. 4:**
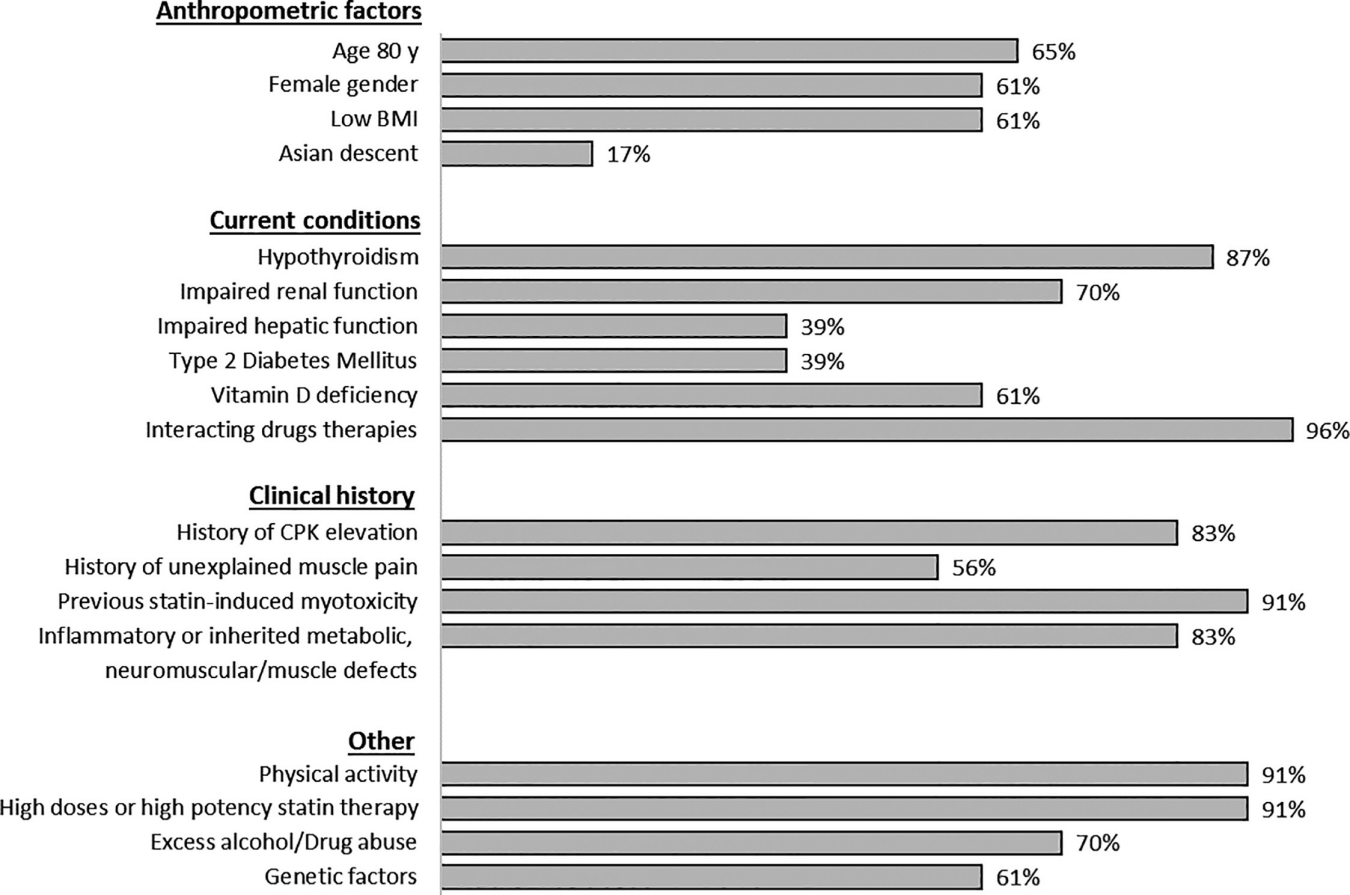
Factors for the development of SAMS considered by lipid specialists in normal clinical practice.

In the differential diagnosis routinely performed by each site, only 17% reported to use validated questionnaires for the evaluation of drug‐event causality in SAMS, but all the lipidologists agreed in declaring the importance of investigating the risk factors for the development of SAMS (Fig. 4). In particular, 65% considered the older age as relevant, 61% female sex or low BMI, 17% Asian ethnicity. The role of physical activity is taken into account in 91%. Hypothyroidism or vitamin D deficiency was considered by 87% and 61%, respectively. The concomitant therapy with potential interacting drugs was evaluated by 96%, whilst a previous experience of statin intolerance by 91%. Discontinuation of statin therapy is reported as a common step following the reporting of SAMS in 91%, and in two cases out of three, it is requested by the same patient.

The therapeutic approach in the rechallenge phase showed a higher use of low‐dose rosuvastatin, pravastatin and fluvastatin, compared with the initial choice (Figure S4). Combination with ezetimibe increased from 14.0% to 33.1%.

## Discussion

The PROSISA study addressed the prevalence of reported SAMS by the lipid specialists in 23 centres and reports a real‐life clinical approach in patients with SAMS by selected Lipid Clinics in Italy. In our study, SAMS were reported by 9.6% of the cohort. This percentage is lower as compared to those reported in other settings. For example, observational studies conducted in the primary care setting reported SAMS in up to 20%–30% of subjects receiving statins [16, 17, 18, 19]. These data suggest the importance of specific education and competence of both healthcare professionals and patients in the management of statin therapy [20]. Notably, the survey responses indicated that the approach to diagnosis and management of SAMS was extremely variable amongst centres, and often not in line with the indications of the current guidelines, even in this setting of specialist centres. However, as already reported in the literature, our estimate of SAMS in a real‐world setting is higher than the incidence rate reported in RCTs [14, 21]. Although the nocebo effect may be relevant, a likely explanation of our results is that the real‐life population receiving statin therapy is remarkably different, that is multiple comorbidities, poly‐pharmacotherapy, lack of motivation and poor adherence, as compared to subjects typically enrolled in RCTs. Moreover, some RCT designs provide a run‐in period in which patients are statin treated, and if intolerant they are excluded from the trial, thereby reducing the final number of adverse drug reactions.

However, the evaluation of the prevalence of muscle‐related signs and symptoms during statin treatment is challenging [9]. Nowadays, the true incidence is unknown: clinicians lack effective biomarkers and tests to confirm diagnosis [5]. The majority of patients reporting SAMS do not present plasma CK elevation, making CK not a reliable nor a clinically meaningful biomarker to properly diagnose statin intolerance [22], whilst a pretreatment increased CK is an indication to search for primary myopathy, although there may be other reasons for an increase in CK, such as intense physical activity. Moreover, it is difficult to get rid of the supposed nocebo effect underlying a vast proportion of reported SAMS [12]. Recent studies suggest that only 30%–50% of patients with self‐reported statin myalgia, as highlighted by a careful clinical examination of the patient, actually experience muscle pain because of statins and not from other causes, highlighted by a careful clinical examination of the patient that may be indeed associated with muscle symptoms. A large portion of supposed statin muscle side effects are likely nonspecific, and several confounders make the diagnosis and treatment of statin myalgia difficult [13, 23, 24]. Accordingly, slightly more than one third of PROSISA patients complaining of SAMS still reported SAMS after dechallenge/rechallenge. However, it should be pointed out that, beside the confirmation of the diagnosis, physicians often deal with patients who complain of discomfort during the treatment and who asks to stop it [6, 25] (or sometimes discontinue the treatment spontaneously, without consulting the physician) [20]. A lack of ‘shared decision‐making’ seemed quite common amongst current statin users [6, 26], and this represents a critical point to be addressed to enhance not only statin adherence, but also patient satisfaction and quality of life on statin therapy [20, 27].

In our study, we also evaluated the impact of patients’ characteristics on the probability of reporting SAMS. The results confirmed the reported role of female gender and of physical activity as risk factors for developing muscle symptoms during statin treatment [28, 29].

We also found that patients aged 65 years or more were less likely to report SAMS. A meta‐analysis of trial conducted by Iwere and Hewitt [30] suggested that the risk of myopathy and rhabdomyolysis was not significantly different in the same age groups, regardless of whether they were exposed to statin monotherapy or a placebo. Thus, it is possible that the reduction in risk we observed in elderly depends on the fact that these patients, characterized by comorbidities and by a worse pathological status, are more aware of the importance of the therapy [31] and less prone to report SAMS. Also, it is possible that the potential of drug–drug interactions in this poly‐treated population leads the physicians to use a lower starting statin dose in older patients, as also recommended by recent guidelines [15], contributing to the results observed in our study.

In our study, the presence of diabetes was associated with a lower probability of reporting SAMS. This should be interpreted in the light of the observational and retrospective nature of the PROSISA study; moreover, we should acknowledge that in the PROSISA cohort, diabetic subjects showed a higher male/female ratio and a higher prevalence of treatment with high‐intensity statins and with potential interacting drugs (both associated with a lower probability of reporting SAMS in our analysis), although the model was adjusted for these covariates. Diabetic patients may be more aware of the importance of statin therapy [25], therefore more willing to tolerate mild muscle symptoms or less prone to develop the nocebo effect [32]. Nevertheless, this result is of potential interest. There is no clear evidence in the literature about the role of diabetes, or any pathophysiological mechanisms associated with the disease, in the incidence of SAMS [28, 33]. The presence of different degrees of peripheral neuropathy [34] might contribute to the lower probability of reporting SAMS in patients with diabetes; however, additional research is needed to confirm the findings also in different populations.

Of note, unlike previously published studies, our analysis modelled the likelihood of reporting SAMS, regardless of whether they were confirmed or not, either by dechallenge/rechallenge or through differential diagnosis and clinical evaluation. This may also explain the results relating to the odds ratios associated with the use of high‐intensity statins or potentially interacting drugs. Indeed, prescription of high‐intensity statins was associated with a lower probability of reporting SAMS (−21%). It is plausible that patients treated with more intense statin therapy are more educated and motivated due to their higher CVD risk, aware of the benefit arising from the statin treatment and less likely to report SAMS. It should be also taken into account that these patients were referred and seen in a specialized network of lipid clinics, which makes this population somewhat selected by the referring physician, who may have decided to consult a specialist for a patient difficult to manage, for example due to the manifest intolerance to statin therapy.

Our study has some limitations that need to be considered whilst interpreting the results. Since the study entails a retrospective and multicentre data collection, the diagnostic criteria for SAMS were neither standardized nor homogeneous between the centres, but rather based on patient reporting and/or on investigator judgement, and the evaluation of some factors was impossible due to the high number of missing data, for example the measurement of CK levels (CK levels were reported in 11% at baseline and in 62% during statin treatment). Finally, it should be remembered that our aim was to evaluate the prevalence of SAMS reported by the patient in a real‐life setting and evaluate any characteristics associated with a higher or lower probability of reporting SAMS. The study design does not allow to evaluate the role of these characteristics as risk factors for confirmed SAMS: this should be addressed through a prospective evaluation, with uniform and standardized criteria of diagnosis and confirmation. Nevertheless, to the best of our knowledge, the PROSISA study is the largest study to have assessed the SAMS‐reporting rate in a real‐life setting. This provides important evidence to improve statin therapy management.

In summary, the PROSISA study allowed to determine the prevalence of statin intolerance due to the appearance of musculoskeletal symptoms in subjects referring to specialized centres. This rate, together with its decrease following dechallenge/rechallenge, highlights the need for prompt, accurate and shared management of statin therapy. Indeed, the PROSISA is the largest study so far highlighting the following relevant clinical points. First, even in highly specialized lipid clinics, the implementation of the approach suggested by the current guidelines in case of SAMS is sketchy and far from adequate, which might imply that amongst nonspecialists and general practitioners, the real‐life approach to SAMS is probably even more inadequate. Secondly, the currently suggested approach to SAMS, which implies a dechallenge/rechallenge pathway, is effective in a real‐life setting and possibly the best option so far since we are missing a clinically relevant biomarker of SAMS; by adopting the dechallenge/rechallenge approach, more than 2/3 of patients initially classified with SAMS were able to receive a statin, resulting in a far better CVD prevention management for these subjects.

In conclusion, the PROSISA study reinforces the strong need to implement the dechallenge/rechallenge approach, both amongst general practitioners and specialists, to provide a far better CVD prevention management in patients initially reported as statin intolerant due to SAMS. Moreover, the study shed some light from a real‐life observation, supporting the good safety profile of statin therapy amongst high‐intensity statin users, elderly subjects and those receiving multiple drug therapy, suggesting that these groups were likely to be highly motivated and educated by general practitioners/specialists, who should provide the proper counselling and support to all treated patients, avoiding premature and unnecessary interruption of a life‐saving pharmacological approach.

## Funding information

The PROSISA study is an initiative of the SISA Foundation supported by an unconditional research grant from Amgen Inc. The authors received no financial support for the research, authorship and/or publication of this article. The work of M Casula is supported by Ministry of Health‐IRCCS MultiMedica GR‐2016‐02361198 and Fondazione SISA. The work of M Arca is supported by Telethon GGP14066; Fondazione Roma Grant 2018‐H2217; National Institute of Health (USA), NIH R01‐HL 131961; Progetti Ricerca Ateneo, RM11715C683968E; and Grandi Progetti Unversità C26H15ZWC9, Fondazione SISA. The work of A Zambon is supported by Fondazione SISA. The work of AL Catapano has been supported by Ministry of Health ‐ Ricerca Corrente ‐ IRCCS MultiMedica, PRIN 2017H5F943 and ERANET ER‐2017‐2364981.

## Conflict of interest statement

M Casula, M Gazzotti, F Bonaiti and E Olmastroni report no disclosures. M Arca has received research funding and/or honoraria for advisory boards or speaker bureau from Alfasigma, Amryt, Amgen, Akcea/Ionis, Boehringer, Daichi‐Sankio, Novartis, Pfizer, Regeneron and Sanofi. M Averna received research funding and/or honoraria for advisory boards, consultancy or speaker bureau from Aegerion, Akcea, Amgen, Merck or MSD, Pfizer and Sanofi‐Regeneron. A Zambon received honoraria/expenses from Amgen, Eli Lilly, Abbott‐Mylan, Servier, Sanofi‐Regeneron, Amryt, Amarin and Daiichi Sankyo. AL Catapano reports grants from Amgen, Sanofi, Regeneron, personal fees from Merck, Sanofi, Regeneron, AstraZeneca, Amgen, Novartis, outside the submitted work.

## Author contribution

**Manuela Casula:** Data curation (equal); Methodology (lead); Supervision (equal); Writing‐original draft (lead). **Marta Gazzotti:** Data curation (equal); Investigation (lead); Methodology (supporting); Supervision (equal); Writing‐original draft (supporting). **Federica Bonaiti:** Data curation (equal); Investigation (supporting); Supervision (supporting); Writing‐original draft (supporting). **Elena Olmastroni:** Formal analysis (lead); Writing‐original draft (supporting). **Marcello Arca:** Conceptualization (supporting); Funding acquisition (supporting); Writing‐review & editing (supporting). **Maurizio Averna:** Conceptualization (supporting); Funding acquisition (supporting); Writing‐review & editing (supporting). **Alberto Zambon:** Conceptualization (lead); Funding acquisition (supporting); Writing‐review & editing (supporting). **Alberico Luigi Catapano:** Conceptualization (supporting); Funding acquisition (lead); Writing‐review & editing (supporting).

## Supporting information

**Figure S1.** Distribution of lipid clinics participating in the PROSISA study network.
**Figure S2.** PROSISA study design.
**Figure S3.** Cumulative percentage of SAMS onset at different time after statin initiation
**Figure S4.** Statin distribution at baseline (*N* = 16 594) and at rechallenge (*N* = 1027).
**Table S1.** Comparison between patients with confirmed and non‐confirmed SAMS (after dechallenge/rechallenge) among subjects reporting symptoms.

## Appendix 1

### PROSISA Study Group*

Local Principal Investigators

Marcello Arca, Anna Montali

Unit of Internal Medicine and Metabolic Diseases, University Hospital Policlinico Umberto I, Rome, Italy

Maurizio Averna, Antonina Giammanco

Promozione della Salute, Materno‐Infantile, di Medicina Interna e Specialistica di Eccellenza (PROMISE), University of Palermo, Italy

Gianni Biolo, Pierandrea Vinci

Clinica Medica, Department of Medical Sciences, University of Trieste, Italy

Claudio Borghi, Sergio D’Addato

Internal Medicine, Sant'Orsola‐Malpighi University Hospital, Bologna, Italy

Antonio Carlo Bossi, Giancarla Meregalli

Endocrine Unit – Diabetes Regional Center, ASST Bergamo Ovest, Treviglio (BG), Italy

Adriana Branchi, Giovanna Squiccimarro

Department of Clinical Science and Community, University of Milan, Ca' Granda IRCCS Foundation, Maggiore Policlinico Hospital, Milan, Italy

Franco Cavalot, Linda Ramadori

Metabolic Diseases and Diabetes Unit, San Luigi Gonzaga University Hospital, Orbassano, Torino, Italy

Francesco Cipollone, Marco Bucci

Clinica Medica Division and European Center of Excellence on Atherosclerosis, Hypertension and Dyslipidemia ‘SS. Annunziata’ Hospital, Chieti, Italy

Maria Del Ben, Francesco Angelico

Internal Medicine I, Policlinico Umberto I, Sapienza University, Rome, Italy

Anna Maria Fiorenza, Emanuela Colombo

Department of Internal Medicine, G. Salvini Hospital, Garbagnate Milanese, Milan, Italy

Liliana Grigore, Veronica Zampoleri

IRCCS Multimedica Hospital, Milan, Italy

Graziana Lupattelli, Vito Gandolfo

Internal Medicine, Department of Medicine, ‘Santa Maria della Misericordia’ Hospital, University of Perugia, Perugia, Italy

Giuseppe Mandraffino, Francesca Savarino

Department of Clinical and Experimental Medicine, Internal Medicine Unit and Lipid Center, G. Martino Hospital, University of Messina, Messina, Italy

Giuliana Mombelli, Chiara Pavanello

Dyslipidemia Center, A.S.S.T. Grande Ospedale Metropolitano Niguarda, Milan, Italy

Livia Pisciotta, Andrea Pasta

Department of Internal Medicine, University of Genoa; Dietetics and Clinical Nutrition Unit, IRCCS‐Policlinic Hospital San Martino, Genoa, Italy

Francesco Purrello, Roberto Scicali

Department of Clinical and Experimental Medicine, Internal Medicine, Garibaldi‐Nesima Hospital, University of Catania, Catania, Italy

Paolo Rubba, Giuliana Fortunato

‘Federico II’ University of Naples, Naples, Italy

Carlo Sabbà, Patrizia Suppressa

Internal Medecine and Geriatrics ‘C. Frugoni’, University of Bari ‘A. Moro’, Bari, Italy

Riccardo Sarzani, Chiara Di Pentima

Internal Medicine and Geriatrics, Department of Clinical and Molecular Sciences, Hospital ‘U. Sestilli’, University ‘Politecnica delle Marche’, Italian National Research Centre on Aging, IRCCS INRCA, Ancona, Italy

Giovanni Battista Vigna, Angela Colangiulo

Internal Medicine, Azienda Ospedaliera‐Universitaria S. Anna, Ferrara, Italy

Josè Pablo Werba, Lorenzo Maria Vigo

Atherosclerosis Prevention Unit, Centro Cardiologico Monzino, IRCCS, Milan, Italy

Sabina Zambon, Lorenzo Previato

Department of Medicine‐DIMED, University of Padua, Padua, Italy

Maria Grazia Zenti, Chiara Maneschi

Division of Endocrinology, Diabetes and Metabolism, Department of Medicine, University Hospital of Verona, Verona, Italy
