# Supplementary material for: Reported muscle symptoms during statin treatment amongst Italian dyslipidaemic patients in the real‐life setting: the PROSISA Study
Source: J Intern Med. 2020 Dec 29;290(1):116–28. doi: 10.1111/joim.13219 (PMC8359216; doi:10.1111/joim.13219)
Supplement: Supplementary file 1 — Figure S1. Distribution of lipid clinics participating in the PROSISA study network. Figure S2. PROSISA study design. Figure S3. Cumulative percentage of SAMS onset at different time after statin initiation Figure S4. Statin distribution at baseline (N = 16 594) and at rechallenge (N = 1027). Table S1. Comparison between patients with confirmed and non‐confirmed SAMS (after dechallenge/rechallenge) among subjects reporting symptoms. [file JOIM-290-116-s001.docx]

**REPORTED MUSCLE SYMPTOMS DURING STATIN TREATMENT AMONGST ITALIAN DYSLIPIDAEMIC PATIENTS IN THE REAL-LIFE SETTING: THE PROSISA STUDY**

**AUTHORS’ NAMES**

Manuela Casula^a,b^, Marta Gazzotti^a^, Federica Bonaiti^a^, Elena OImastroni^a^, Marcello Arca^c^, Maurizio Averna^d^, Alberto Zambon^b,e^, Alberico L. Catapano^a,b^, on behalf of the PROSISA Study Group*

**AFFILIATIONS**

a Epidemiology and Preventive Pharmacology Service (SEFAP), Department of Pharmacological and Biomolecular Sciences, University of Milan, Milan, Italy

b IRCCS MultiMedica, Sesto S. Giovanni (MI), Italy

c Department of Translational and Precision Medicine, Unit of Internal Medicine and Metabolic Diseases Sapienza University, Rome, Italy

d Department of Health Promotion Sciences Maternal and Infantile Care, Internal Medicine and Medical Specialities, University of Palermo, Palermo, Italy

e Department of Medicine-DIMED, University of Padua, Padua, Italy

**SUPPLEMENTARY MATERIAL**


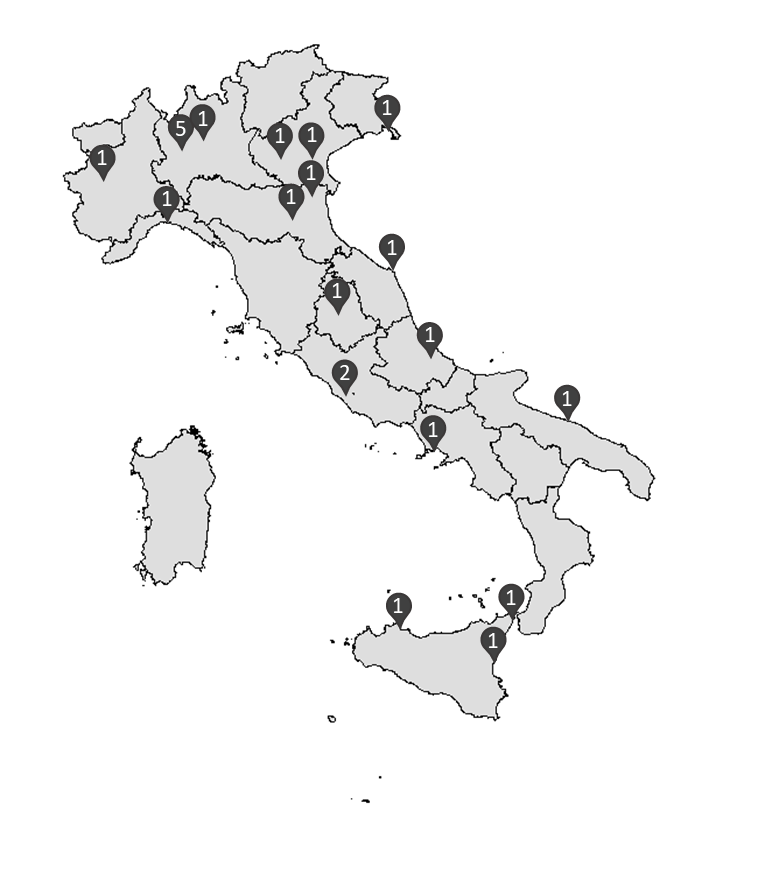


**Supplementary Figure 1**. Distribution of lipid clinics participating in the PROSISA study network


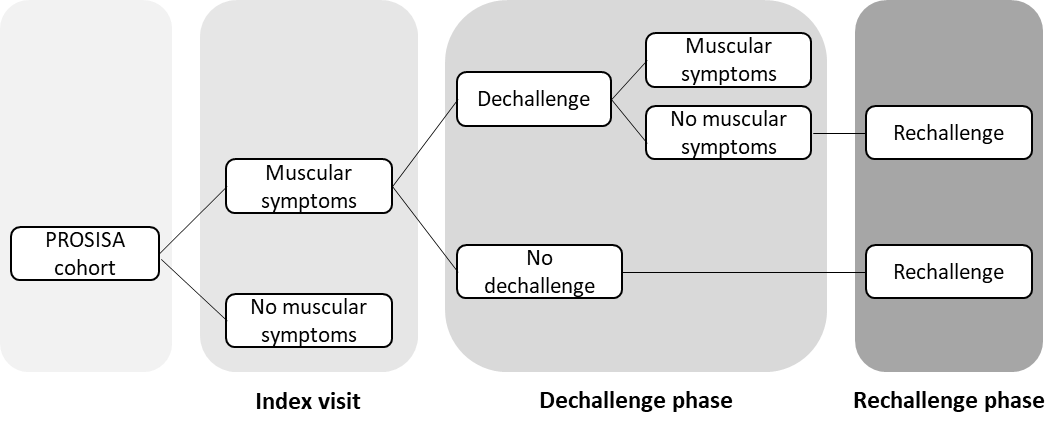


**Supplementary Figure 2.** PROSISA study design


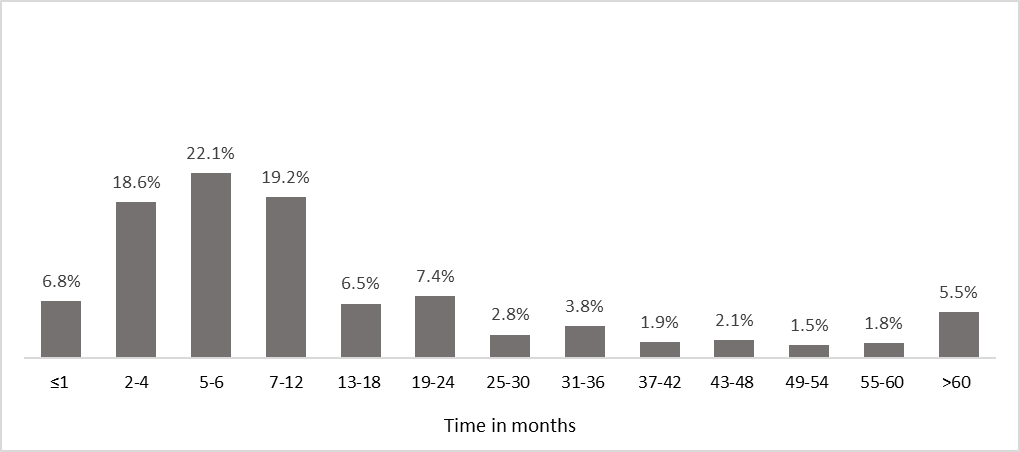


**Supplementary Figure 3.** Cumulative percentage of SAMS onset at different time after statin initiation

**Supplementary Table 1**. Comparison between patients with confirmed and non-confirmed SAMS (after dechallenge/rechallenge) among subjects reporting symptoms

|  | **non-confirmed SAMS (N = 810)** | **confirmed SAMS  (N = 504)** | **P value** | **TOTAL** |
| --- | --- | --- | --- | --- |
| **Male gender** | 46.2% | 46.0% | .524 | 48.0% |
| **Age >= 65** | 33.5% | 34.8% | .550 | 31.0% |
| **Physical activity** | 20.6% | 14.3% | .012 | 25.6% |
| **Interacting drugs** | 5.2% | 2.2% | .006 | 2.7% |
| **Hypertension** | 49.6% | 49.0% | .500 | 53.3% |
| **Type 2 diabetes mellitus** | 13.0% | 13.3% | .313 | 15.1% |
| **Any previous cardiovascular event** | 30.1% | 32.9% | .127 | 32.2% |

**
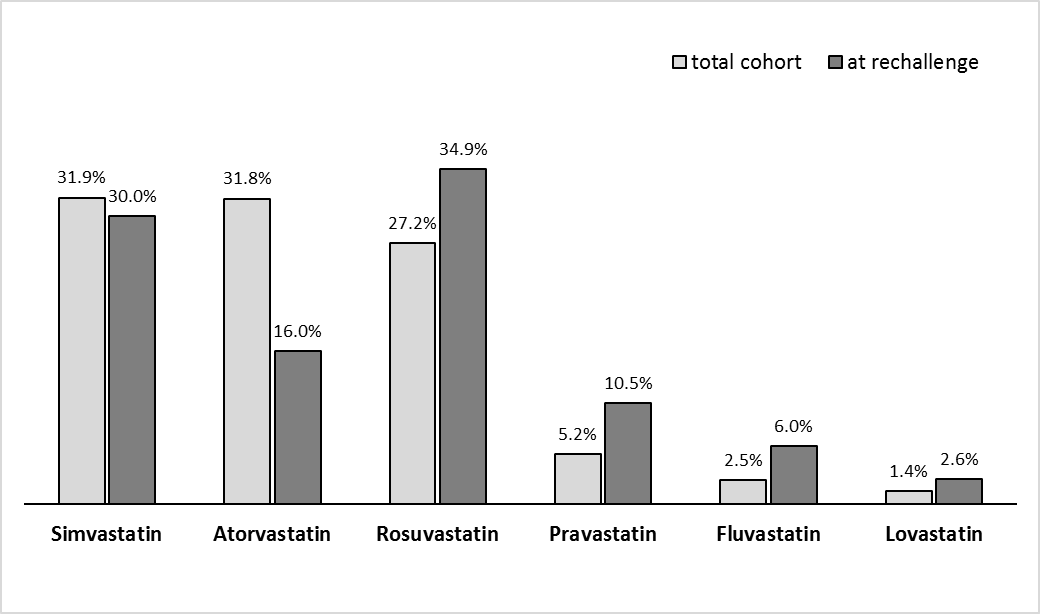
Supplementary Figure 4.** Statin distribution at baseline (N=16,594) and at rechallenge (N=1,027)
